# Research trends between childhood obesity and gut microbiota: a bibliometric analysis (2002–2023)

**DOI:** 10.3389/fmicb.2024.1461306

**Published:** 2024-09-27

**Authors:** Mengping Wang, Zhen Zhang, Yuxuan Liu, Enlin Jian, Peng Ye, Hongjie Jiang, Xiaoping Yu, Peiling Cai

**Affiliations:** ^1^School of Preclinical Medicine, Chengdu University, Chengdu, China; ^2^Clinical Medical College and Affiliated Hospital of Chengdu University, Chengdu, China

**Keywords:** children, obesity, gut microbiota, bibliometric analysis, hotspots and research trend

## Abstract

**Background:**

In recent years, the prevalence of childhood obesity has escalated alarmingly, posing significant threats to the physical and mental well-being of children, with an elevated likelihood of persisting into adulthood. Notably, recent investigations have uncovered a profound association between intestinal microbiota, a crucial component of the internal milieu, and childhood obesity. Disturbances in intestinal microbiota and their by-products are now understood to be profoundly intertwined with the evolutionary pathway of childhood obesity. Bibliometric analysis offers a deep understanding of the current research landscape, so we apply it to a review of the emerging trends and patterns between childhood obesity and gut microbiota.

**Materials and methods:**

We conducted a rigorous and extensive search of the Web of Science (WoS) Core Collection database, spanning the years from 1900 to 2023, to analyze scholarly articles pertaining to childhood obesity and gut microbiota. Utilizing VOSviewer, CiteSpace, the R package “bibliometrix,” and the online bibliometric analysis platform (https://bibliometric.com/), we delved into the intricate details of research hotspots, academic collaborations, and emerging trends within this domain.

**Results:**

The exhaustive search encompassed the globe, uncovering a cumulative total of 1,384 pertinent studies originating from 429 nations. The results were compelling, revealing a profound influence exerted by the United States and China in this specific field of research. Furthermore, it was observed that the volume of scholarly works pertaining to childhood obesity and gut microbiota is steadily growing year on year. The current hot topics in this field include “abuse,” “maltreatment,” “adverse childhood experiences,” “students,” and “food addiction”.

**Conclusion:**

This comprehensive review offers a meticulous exploration of the evolving trends and emerging research agendas pertaining to childhood obesity and gut microbiota over the past two decades. It strives to equip researchers with a thorough understanding of the key nations, institutions, journals, and potential collaborators in these specialized fields. Additionally, it sheds light on the current frontiers of research and strategic avenues for further exploration, thus serving as an invaluable resource for scholars delving deeper into the intricacies of childhood obesity and the gut microbiome.

## 1 Introduction

Obesity is a major public health problem (Abdallah Ismail et al., 2011). Globally, an alarming figure of over 39% of the total population falls under the obese category (Lee et al., 2024). What’s more, obesity is not just a phenomenon affecting individuals of all ages, particularly the younger generations, but its associated comorbidities pose a considerable threat to both physical and mental wellbeing (Pan et al., 2021). The escalating trend of obesity in younger age groups has drawn our attention to the pediatric population, where obesity rates are soaring (Jeewandara et al., 2024).

The worldwide surge in childhood obesity rates has sparked widespread concern regarding the health outcomes of individual children and the urgency for robust health systems (Slighting et al., 2024; Aslantas et al., 2024). Childhood obesity is a grave health concern that plagues children and adolescents aged 5–19 years globally (Tian et al., 2024). This condition is closely linked to an augmented risk and premature manifestation of non-communicable diseases, including type 2 diabetes mellitus (La Grasta Sabolic et al., 2024), a spectrum of cancers such as colorectal and breast cancers (Weihrauch-Blüher et al., 2019), and cardiovascular diseases (Huang et al., 2024). Childhood obesity not only has negative psychosocial effects but also impacts eating and sleeping habits, thereby reducing quality of life (NEJM, 2024). This condition often persists into adulthood, increasing the risk of various non-communicable diseases (NCDs) (Guaresti et al., 2024). Given that childhood obesity predicts adult obesity, it is crucial to address this issue early to mitigate both current and future harms (Ajala et al., 2017). The etiology of childhood obesity is multifaceted, influenced by various factors including family dynamics (Ding et al., 2024), genetic predispositions (Chodick et al., 2024), and environmental elements such as sleep patterns, dietary choices, physical activity levels, and socioeconomic status (Richter et al., 2024).

The gut microbiota, a distinct group of microorganisms within the human body, plays a crucial role in maintaining health (Wang et al., 2024a). Recent studies have demonstrated the critical influence of gut microbiota–a significant component of the internal ecosystem–on childhood obesity, highlighting that disruptions in gut microbiota are characteristically observed in obese children (Peng L. J. et al., 2024). The association between intestinal microbiota and childhood obesity has emerged as a scientific domain garnering considerable attention in recent years (Tang et al., 2024). An increasing number of studies indicate a strong correlation between intestinal microbiota and childhood obesity. Disturbances in intestinal microbiota and their metabolites, including short-chain fatty acids, bile acids, indoles, and their derivatives (Wang et al., 2020; Li Q. et al., 2023), are implicated in the onset of childhood obesity (Fonvig et al., 2014). Conversely, dietary quality, sleep patterns, and physical well-being can indirectly impact the gut microbiota, leading to metabolic irregularities that foster the progression of obesity in children (Gunawan et al., 2024; Caserta et al., 2023). Comorbidities associated with childhood obesity, such as type 2 diabetes and metabolic disorders, exhibit a close association with the gut microbiota (Gong et al., 2024). Research reveals notable differences in the gut microbiota between obese children and their normal-weight counterparts (Da Silva et al., 2020; Li et al., 2024). Notably, intestinal microbiota constitutes a diverse and intricate community of microorganisms residing in the human gastrointestinal tract over extended periods. Predominantly, this ecosystem is characterized by two major phyla: the anaplasma phylum and the thick-walled phylum, collectively representing roughly 90% of the composition. This profile aligns closely with the adult intestinal microbiota from the age of three in children (Jandhyala et al., 2015). Currently, the ratio between the thick-walled phylum and the anabolic phylum is considered a potential indicator of childhood obesity (Krajmalnik-Brown et al., 2012). Obese children exhibit diminished levels of dominant gut microbiota (Cross et al., 2017) and a lower count of advantageous bacteria (Li et al., 2024). These variations might lead to disparities in energy harvesting, lipid accumulation, and metabolic processes in obese children (Luzzi et al., 2024). Alterations in the composition and relative diversity of the gut microbiota correlate with weight status during early childhood (Mbakwa et al., 2018). Obese children demonstrate diminished gut microbial diversity, suggesting a less stable gut milieu (Morgado et al., 2023), impacting energy metabolism and utilization. Cutting-edge microbiome research has uncovered a linkage between the gut microbiome and the brain, indicating a reciprocal communication pathway along the gut-microbiome-brain axis in obesity’s pathophysiology (Lane et al., 2023). A hypothesis explaining the correlation between childhood obesity and neurological changes suggests that these brain variations predate excessive weight gain, acting as a predictive factor for future weight increase and offering insights into the primary drivers of this process. Alternatively, these neural discrepancies might arise subsequent to weight gain, reflecting the secondary impacts of metabolic dysfunctions or other physiological repercussions aggravated by obesity (Carnell, 2024). At present, numerous investigations have established a causal link between an imbalance in gut microbiota and childhood obesity (Riva et al., 2017). However, a comprehensive understanding of the underlying mechanisms remains elusive, necessitating expedited discovery and thorough examination of the causative pathways connecting gut microbiota and childhood obesity. Such insights are vital for refining intervention strategies and fostering the advancement of novel treatments.

Notwithstanding advancements in research on childhood obesity and gut microbiota, numerous queries persist warranting further exploration. Hence, conducting a quantitative evaluation of prevailing research emphases, focal areas, and prospective trajectories in this domain is essential. Bibliometrics employs mathematical and statistical approaches to quantitatively assess scholarly publications, examining their framework, attributes, and developments over time (Cheng et al., 2022a; Wu et al., 2022; Su et al., 2023). Integral to the information sciences, it elucidates the principles governing scholarly content and its curation (Cheng et al., 2022b; Zhu et al., 2022). By quantitatively scrutinizing the literature and comprehending the field’s evolution, one can unravel scientific endeavor patterns and leverage assorted bibliometric measures to steer future investigative endeavors effectively. This manuscript marks the pioneering systematic visualization of the childhood obesity-gut microbiota interface through bibliometric techniques. Encompassing the period from January 1900 to December 2023, we thoroughly employed bibliometric methodologies to delve into the profound advancements in research concerning childhood obesity and gut microbiota, aiming to discern prevailing investigative domains, prevalent topics, and impending trajectories.

## 2 Materials and methods

### 2.1 Data source

The Web of Science (WoS) Core Collection stands as a comprehensive and impeccably current database, a beacon of academic excellence spanning across over 190 disciplines worldwide (Han et al., 2023; Petermann-Rocha et al., 2024). It houses an extensive repository of over 12,000 renowned and influential scientific journals, serving as a testament to its profound scholarly impact. Acknowledged as the epitome of bibliometric research across disciplines, WoS Core Collection offers unparalleled services in literature exploration and citation analysis, while ensuring real-time data updates (Aggarwal et al., 2016; Jiang et al., 2018).

### 2.2 Data acquisition

**Figure 1:**
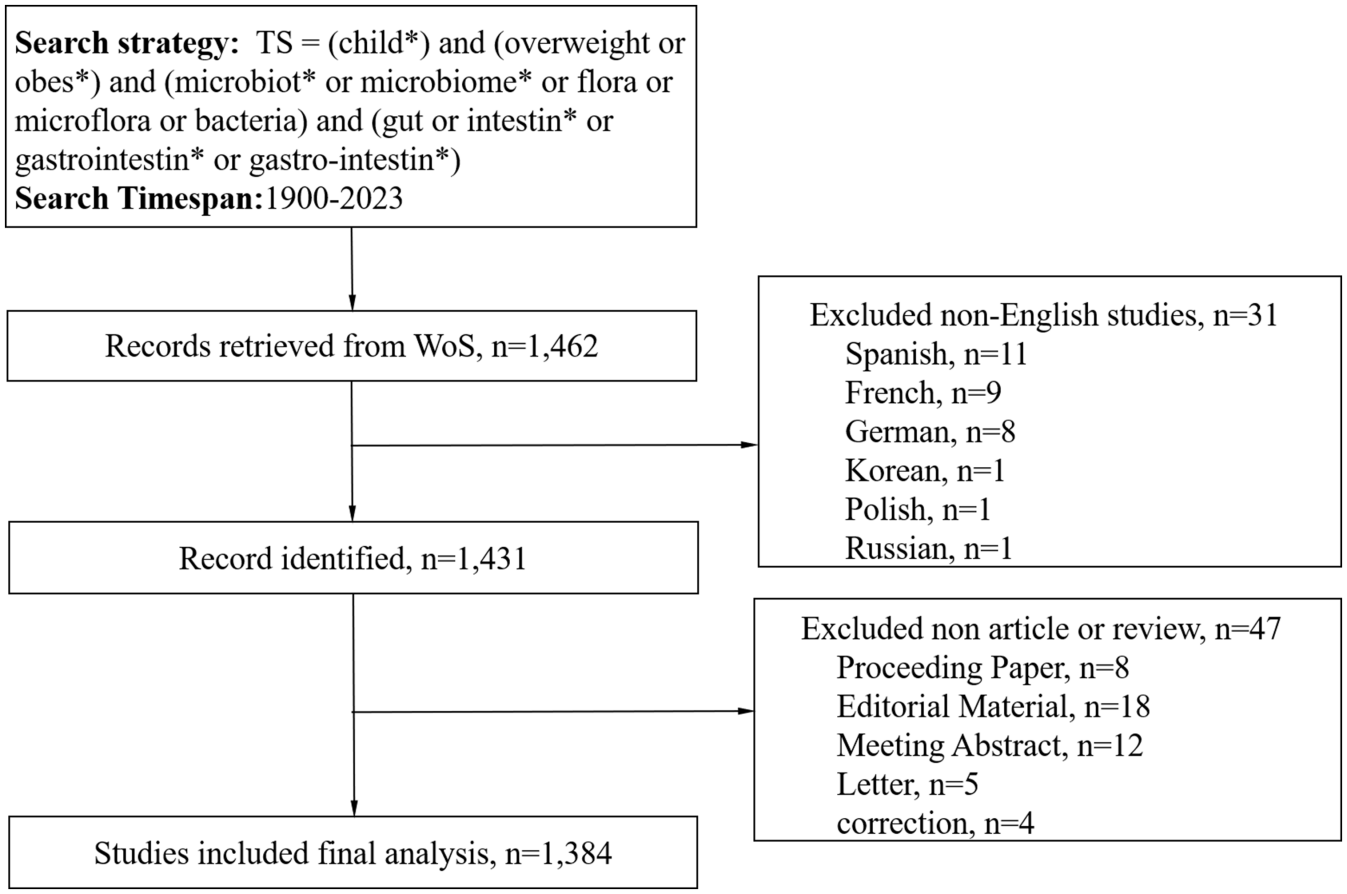
Flowcharts of the publication selection.

A diligent literature search was conducted in the WoS Core Collection, spanning the vast timeline from January 1, 1900, to December 31, 2023. This search was executed on February 5, 2024, with the intention of capturing the essence of scholarly works within the specified period. The retrieved publications were downloaded in plain text format, ensuring a streamlined and accessible database for subsequent scrutiny. The search methodology incorporated an array of pivotal terms and combinations: TS = (child*) AND TS = (overweight OR obes*) AND TS = (microbiot* OR microbiome* OR flora OR microflora OR bacteria) AND TS = (gut OR intestin* OR gastrointestin* OR gastro-intestin*). The scope of this search was narrowed to English-language publications, encompassing only articles or review documents. Meanwhile, we included only publications from 2023 and earlier, since there was fewer publication in 2024 as of the search data to form combining results when conducting bibliometric analysis. The methodology employed in the selection and analysis of these articles was visually shown in Figure 1.

### 2.3 Bibliometric analysis

A meticulous bibliometric analysis was conducted utilizing VOSviewer, the robust R package “bibliometrix” within the esteemed R software, CiteSpace, and the comprehensive online bibliometric analysis platform. This extensive study harnessed the power of VOSviewer and CiteSpace to delve into the intricate country collaboration heat maps, institutional collaboration networks, intricate author and co-citing author collaboration networks, comprehensive co-citation reference timeline maps, and revealing keyword burst maps. Furthermore, annual publication trends, intricate national collaboration networks, detailed author publications, and insightful journal publication trends were carefully constructed using the versatile R package “bibliometrix” and the aforementioned online bibliometric analysis platform.^1^

All raw data used in this study were obtained from public databases, therefore no ethical review was necessary.

## 3 Results

### 3.1 Analysis of publications

**Figure 2:**
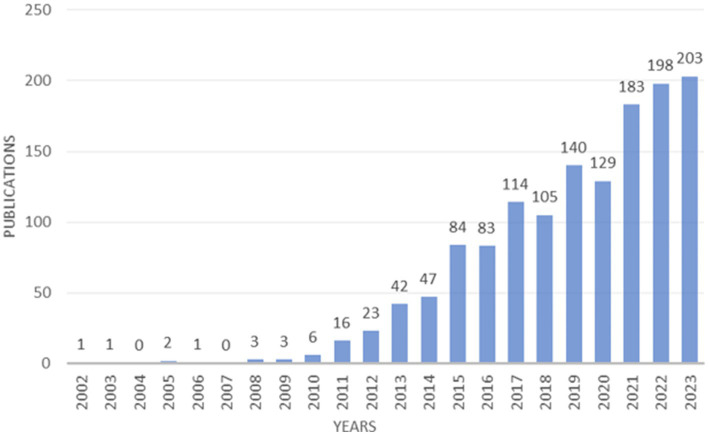
The number of publications related to childhood obesity and gut microbiota.

Between 2002 and 2023, a comprehensive survey of academic output reveals a significant contribution of 1,384 papers focused on the intricate connection between childhood obesity and gut microbiota. These papers represent an average annual output of 66 studies, indicating a sustained interest in the subject matter. Starting in 2010, there has been a consistent increase in the number of published papers, reaching its peak in 2023 (Figure 2). Prior to 2010, the number of yearly publications hovered at a minimal level, averaging only two papers per year. Strikingly, 2023 marked a pinnacle in this research area, accounting for 203 publications, which constitute a substantial 14.67% of the total number of records. Overall, the increasing trend in the number of publications since 2010 underscores the escalating relevance and vitality of this research domain.

### 3.2 Analysis of countries/regions

**Figure 3:**
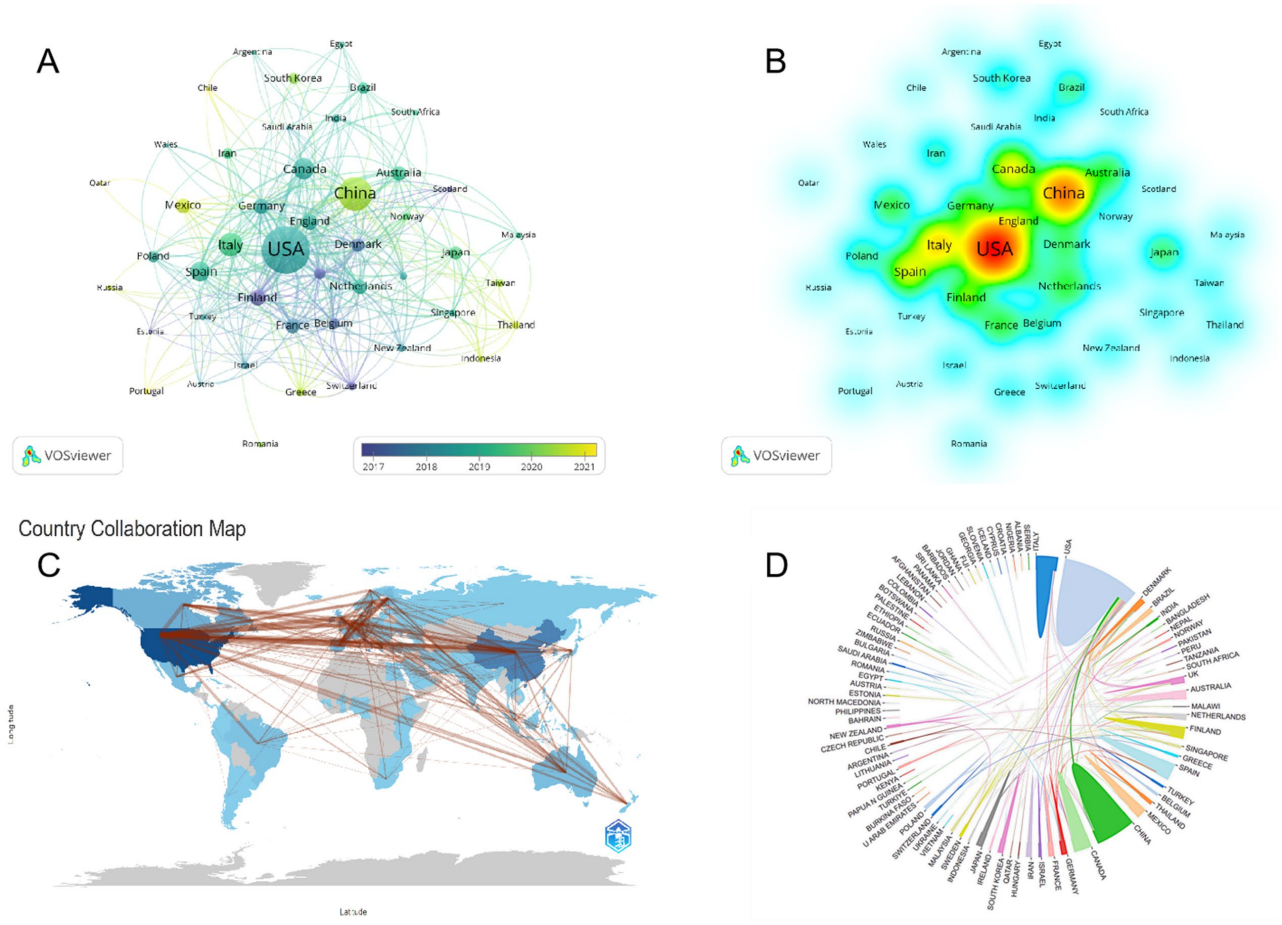
Country cooperation network map. Taiwan is a region of China. **(A)** Visual mapping of country co-author coverage using VOSviewer. The varying node colors in this visualization reflect the average appearance year (AAY) for each country, represented by the color gradient in the lower right corner. **(B)** Density map of country/region postings. As the density increases, indicating a greater number of papers published in that particular country/region, the color transitions toward a deeper red, signifying a higher proportion of the overall publication volume originating from that location. **(C)** Collaborative network shown on the world map. **(D)** The international collaboration among pertinent countries/regions.

The United States American (United States) and China emerged as significant contributors to the research on childhood obesity and gut microbiota (Table 1), accounting for a substantial 48.8% of the total publications, with Canada contributing 7.1% (*n* = 98). Among the 46 participating countries/regions, the top two players, the United States and China, exhibited the highest total literature share (TLS), signifying their preeminent status in this research area. We employed VOSviewer to create a visual representation of national co-authorships, incorporating solely those countries/regions with more than five publications (Figure 3A). Light purple hues signify early involvement in research on childhood obesity and gut microbiota, whereas yellow nodes indicate a more recent entry into this field. We can observe the number of publications per country/region in the density visualization map (Figure 3B). The regions encompassing the United States and China display the darkest hues, closest to red, revealing their paramount weight in terms of both publication volume and number.

**Table 1:** The top 10 countries with the most publications in the realm of childhood obesity and gut microbiota.

Rank	Country	Record Count	% of (1384)	Citations	Total link strength
1	United States	440	31.8%	28,632	236
2	China	235	17.0%	8,561	124
3	Italy	124	9.0%	6,071	92
4	Canada	98	7.1%	5,939	83
5	Spain	86	6.2%	4,425	86
6	Finland	61	4.4%	10,174	86
7	England	58	4.2%	4,294	102
8	Germany	55	4.0%	5,507	103
9	Australia	50	3.8%	3,153	51
10	France	48	3.5%	6,254	85

Regarding global collaboration trends (Figure 3C), research in this domain is primarily concentrated in developed nations. China, as the world’s foremost economy, is likewise actively engaged in this research. United States, China, and Italy lead in terms of the number of publications, while United States and Finland stand out as the most cited countries. Notably, the collaboration between United States and China forms the most significant multicenter network in this research domain (Figure 3D), further emphasizing their pivotal roles in advancing our understanding of childhood obesity and gut microbiota.

### 3.3 Analysis of affiliations

**Figure 4:**
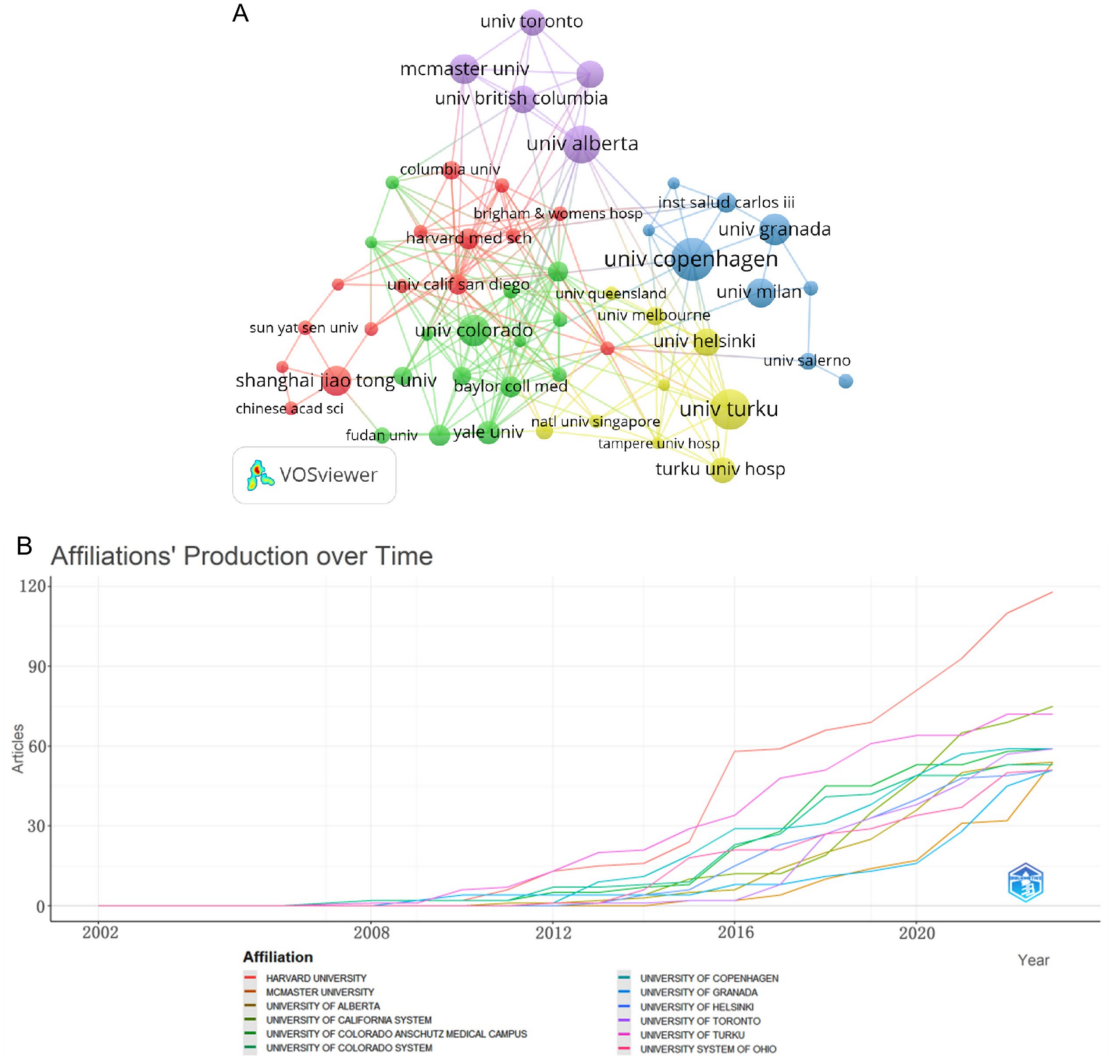
Network diagram of institutions in the field of childhood obesity and gut microbiota. **(A)** Plot of institutional trends in publications over time. **(B)** Inter-institutional collaboration network visualized by VOSviewer.

The intricate inter-institutional collaboration network map serves to vividly illustrate the intricate web of collaborations between various institutions and their respective research areas. The University of Copenhagen stands as the most prolific institution, boasting a total of 34 articles (Table 2). Closely following it, we have the University of Turku and the University of Alberta, each with 32 and 30 articles, respectively. We can see a fascinating insight provided by Figure 4A; almost every institution has embarked on extensive research pertaining to childhood obesity and gut microbiota since 2009, exhibiting a consistent upward trend in this domain. Among these, Harvard University and the University of Turku emerge as leading pioneers in this field.

**Table 2:** The top 10 institutions in terms of the number of publications in childhood obesity and gut microbiota.

Rank	Institution	Country	Publications	Citations	Average citations
1	University of Copenhagen	Denmark	34	5,968	175.53
2	University of Turku	Finland	32	4,703	146.97
3	University of Alberta	Canada	30	2,192	73.07
4	University of Colorado	United States	25	2,825	113.00
5	University of Granada	Spain	25	1,650	66.00
6	McMaster University	Canada	24	1,211	50.46
7	Shanghai Jiao Tong University	China	24	726	30.25
8	University of Milan	Italy	23	741	32.22
9	University of Manitoba	Canada	22	2048	93.09
10	University of British Columbia	Canada	22	1,678	76.27

With a minimum threshold of 10 published articles, the institutions involved in the realm of childhood obesity and gut microbiota research were comprehensively visualized and analyzed utilizing VOSviewer. Upon examination of Figure 4B, it is evident that there are 53 institutions, categorized into five distinct color-coded groups. Notably, the red and green clusters constitute the largest contingents, each comprising 15 entities, among which we find Shanghai Jiao Tong University, Harvard Medical School, Colorado University, and Yale University. These institutions have fostered robust collaborations within their respective clusters, indicating a concentrated focus on research topics that intersect with childhood obesity and gut microbiota. It is noteworthy that all four of the top-tier institutions originate from Canada, a country that holds significant influence in the realm of childhood obesity and gut microbiota research.

### 3.4 Analysis of authors

**Figure 5:**
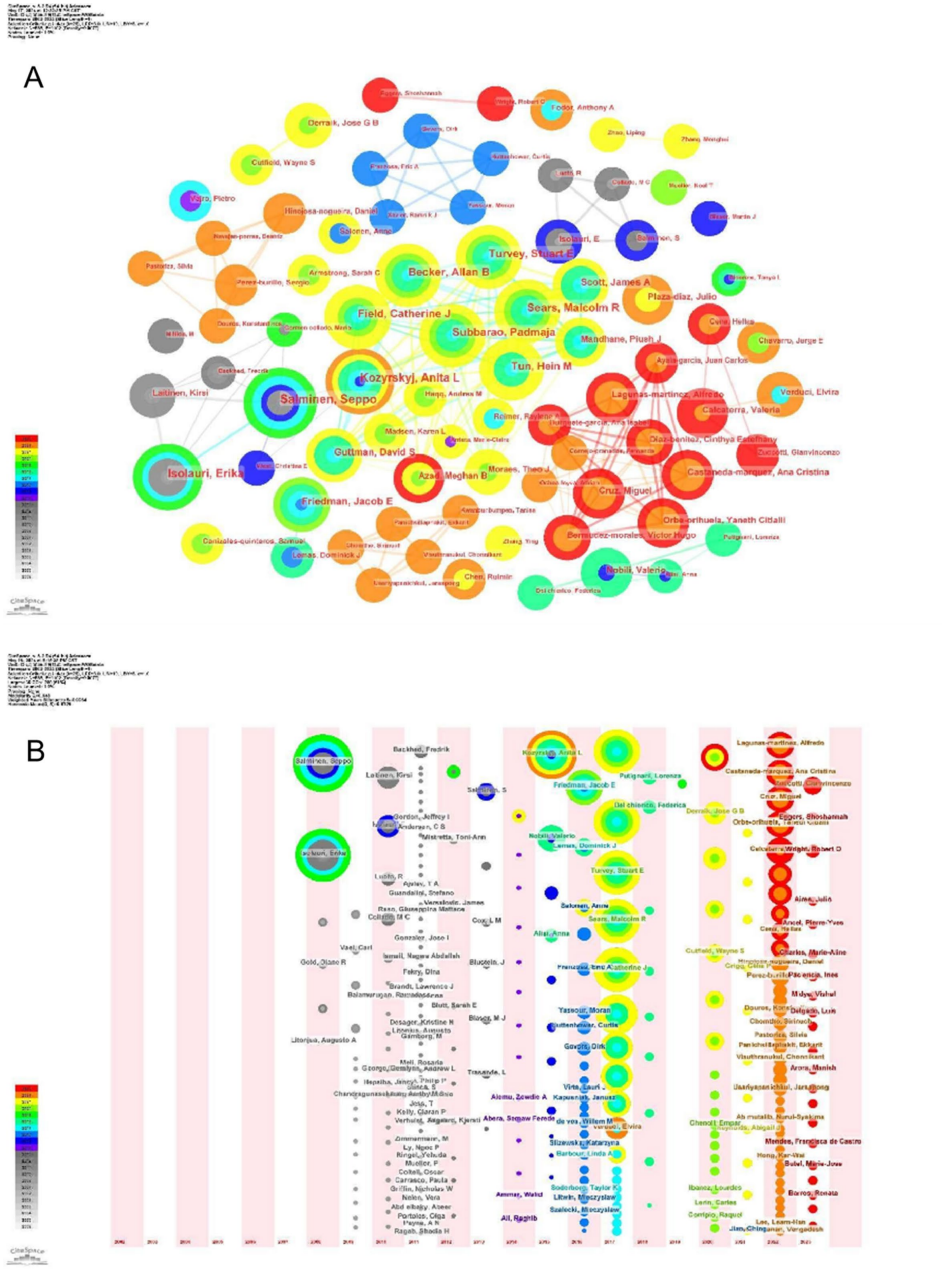
Author analysis. **(A)** Collaboration networks. Each node, distinguished by varying hues and sizes, represents an individual researcher. The size of a node is indicative of the number of publications, whereas the color signifies the year of publication. **(B)** Timeline. The colors of the circles represent the years from 2002.

Over the span of twenty decades, commencing from 2002 to 2023, an impressive tally of 8,076 authors contributed papers exploring the intersection of childhood obesity and gut microbiota. The top 10 most prolific authors in this field, along with related details are listed in the Table 3. These individuals collectively authored 130 papers, accounting for 9% of the total research output. Among these esteemed scholars, Salminen, Seppo from Finland emerged as the preeminent researcher, having dedicated 16 papers to the subject of childhood obesity and gut microbiota. The subsequent two leading authors, Isolauri, Erika of Finland, and Subbarao, Padmaja of Canada, also exhibited remarkable dedication to this field. The CiteSpace visualization portrays an intricate network of authors, who are actively researching the correlation between childhood obesity and gut microbiota (Figure 5A). The network underwent further examination via a timezone view analysis, which exposed the time-based distribution of author contributions (Figure 5B). Among these eminent researchers, Salminen, Seppo and Kozyrskyj, Anita L., stand out for their centrality scores, both scoring an impressive 0.01.

**Table 3:** The top 10 authors with the highest number of publications in childhood obesity and gut microbiota.

Rank	Author	Country	Publications	Citations	Average citations	H-index
1	Salminen, Seppo	Finland	16	2,885	180.31	16
2	Isolauri, Erika	Finland	15	2,955	197.00	17
3	Subbarao, Padmaja	Canada	14	862	61.57	11
4	Turvey, Stuart E.	Canada	14	862	61.57	11
5	Becker, Allan B.	Canada	13	859	66.08	12
6	Sears, Malcolm R.	Canada	12	855	71.25	11
7	Kozyrskyj, Anita L.	Canada	12	977	81.42	13
8	Field, Catherine J.	Canada	12	831	69.25	10
9	Tun, Hein M.	China	11	745	67.73	11
10	Cruz, Miguel	México	11	165	15.00	6

**Figure 6:**
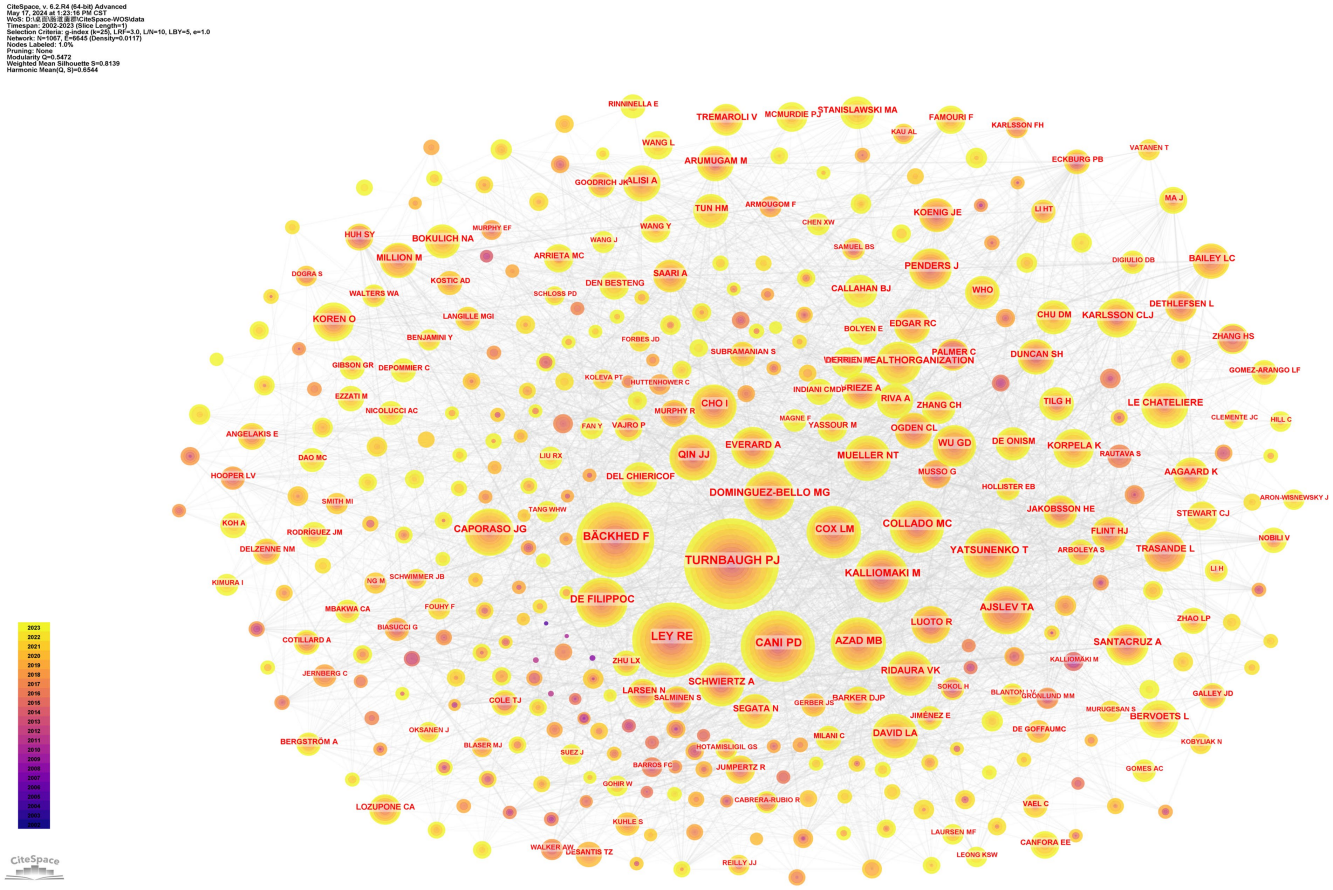
Co-cited authors.

Additionally, information for the top 10 most cited authors and their respective details are compiled in the Table 4. Among these highly cited authors, Turnbaugh, Pj from the United States, Ley, Re, from the United States, and Bäckhed, F from Denmark, occupy the prestigious top three positions in terms of their number of publications. The intricate network displayed in Figure 6 showcases a multitude of prominent authors whose research has accumulated significant citations, offering a captivating visual representation. This network not only reflects the knowledge structure but also highlights the patterns of collaboration among influential researchers in the realm of childhood obesity and gut microbiota research.

**Table 4:** The top 10 cited authors with the highest number of citations in childhood obesity and gut microbiota.

Rank	Cited author	Country	Citation	Total link strength
1	Turnbaugh, Pj	United States	788	20,226
2	Cani, Pd	Belgium	510	13,532
3	Ley, Re	United States	495	12,733
4	Bäckhed, F	Denmark	462	13,433
5	Collado, Mc	Spain	268	8,898
6	Azad, Mb	Canada	248	8,084
7	Cox, Lm	United States	214	6,175
8	Caporaso, Jg	United States	198	4,133
9	Dominguez-bello, Mg	United States	194	6,595
10	Kalliomaki, M	Finland	187	4,950

### 3.5 Analysis of journals

Since the commencement of the decade in 2010, there has been a pronounced surge in the volume of scholarly works dedicated to exploring the intricate relationship between childhood obesity and gut microbiota. This upward momentum is anticipated to continue unabated. These diverse studies have been published across a broad spectrum of 531 journals, with the foremost 15 journals outlined in Table 5. At the forefront, the journal ‘Nutrients’ stands tall with a substantial 75 articles (5.4%), boasting an impressive 419 citations and a robust link strength of 136. It is closely followed by ‘Scientific Reports’ and ‘Plos One’, Both contributing 35 articles (2.5%), ‘Frontiers in Microbiology’ with 33 articles (2.4%), and ‘International Journal of Obesity’ with 30 articles (2.2%). Among these, ‘Gut Microbes’ emerged as the journal with the highest impact factor, boasting an IF of 12.2. As evident from Table 6, the 10 journals that garnered the highest co-citation counts each received over 500 citations. Notably, ‘Nature’ leads the pack with a staggering 3,185 co-citations, closely trailed by ‘PLoS One’ with 2,830, ‘P Natl Acad Sci USA’ with 2074, ‘Am J Clin Nutr’ with 1947, and ‘Int J Obesity’ with 1,696. Among these, ‘NATURE’ reigns supreme with an IF of 64.8, reflecting its profound influence in the field.

**Table 5:** The top 15 journals in terms of the number of publications relating to childhood obesity and gut microbiota.

Rank	Source	Documents	IF	Citations	Total link strength
1	Nutrients	75	5.9	419	136
2	Scientific Reports	35	4.6	168	85
3	Plos One	35	3.7	90	76
4	Frontiers in Microbiology	33	5.2	40	67
5	International Journal of Obesity	30	4.9	74	178
6	Pediatric Obesity	23	3.8	152	101
7	Journal of Pediatric Gastroenterology and Nutrition	22	2.9	197	54
8	Microorganisms	21	4.5	1,529	68
9	Frontiers in Nutrition	17	5	274	24
10	Frontiers in Endocrinology	17	5.2	169	61
11	Frontiers in Pediatrics	16	2.6	82	51
12	Gut Microbes	15	12.2	110	44
13	Pediatric Research	15	3.6	293	38
14	Children-Basel	14	2.4	1,412	28
15	Frontiers in Cellular and Infection Microbiology	14	5.7	359	15

**Table 6:** The top 10 co-cited journals in terms of the number of publications relating to childhood obesity and gut microbiota.

Rank	Sources-R	Citation	Country	IF
1	Nature	3,185	United Kingdom	64.8
2	PLoS One	2,830	United States	3.7
3	P Natl Acad Sci USA	2074	United States	11.1
4	Am J Clin Nutr	1947	United States	7.1
5	Int J Obesity	1,696	United Kingdom	4.9
6	Nutrients	1,631	Switzerland	5.9
7	Science	1,395	United States	56.9
8	Sci Rep-Uk	1,321	United Kingdom	4.6
9	Gut	1,254	United Kingdom	24.5
10	Pediatrics	1,123	United States	8

**Figure 7:**
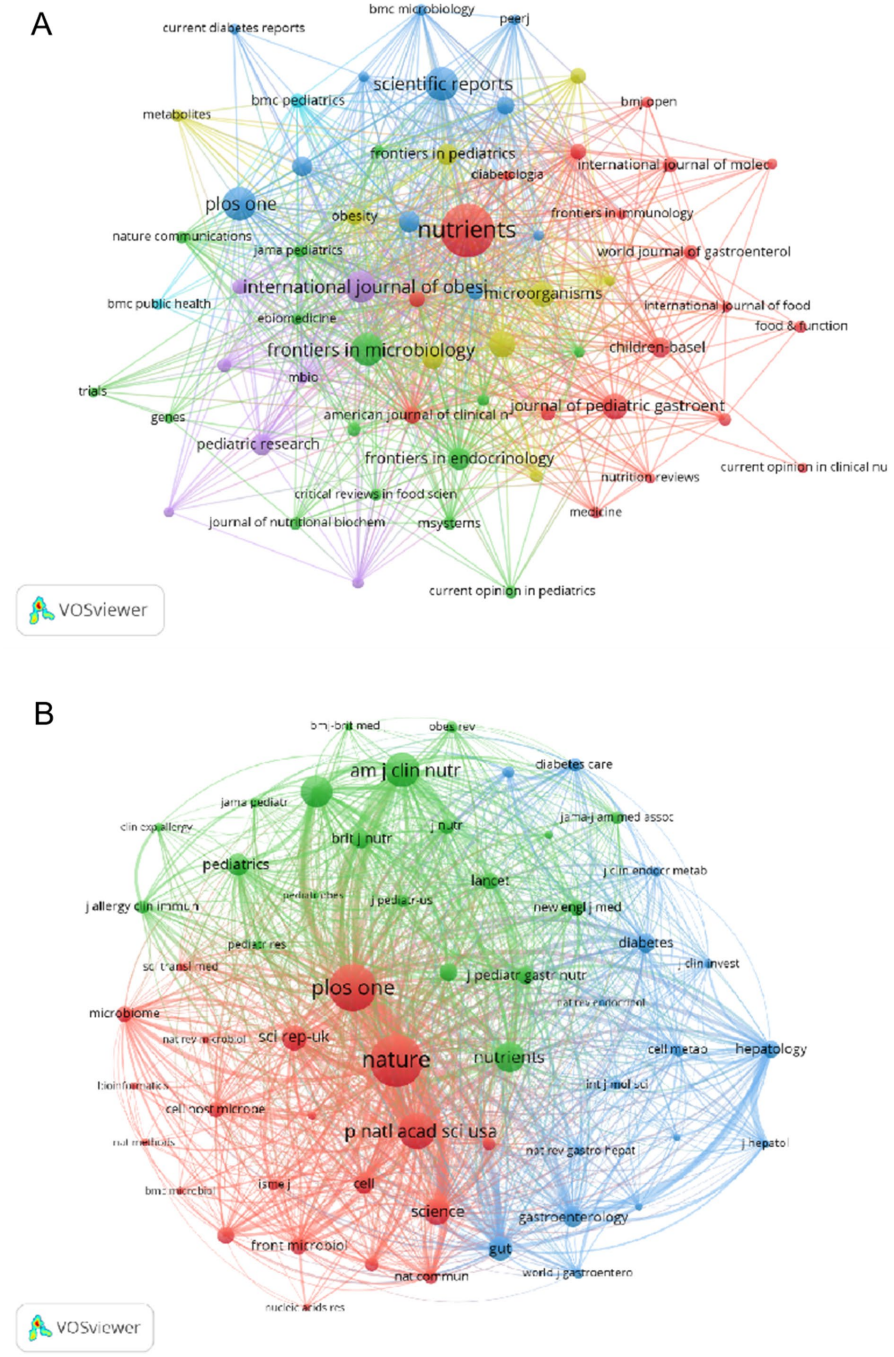
Journals analysis. **(A)** Visualization of Journal Publications in the field of childhood obesity and gut microbiota research. **(B)** Visualization of Co-cited Journals.

Utilizing VOSviewer, we imposed a threshold of a minimum of 5 scholarly articles per journal, resulting in the selection of 63 eligible journals from the initial 531. This dataset was then utilized to construct an intricate journal network diagram, depicted in Figure 7A. It is noteworthy that robust citation linkages emerged within the journals ‘Nutrients. ‘Scientific Reports. ‘PLoS One, ‘Frontiers in Microbiology’, and ‘International Journal of Obesity’. Subsequently, by setting the minimum co-citation threshold at 300, we filtered out 57 journals to construct a co-citation network graph, as illustrated in Figure 7B. In this intricate network, the size of each node corresponds to the number of citations it has received, highlighting the dominance and leadership of ‘Nature, ‘PLoS One, ‘Proc Natl Acad Sci USA’, and ‘Am J Clin Nutr’ in guiding and shaping the research landscape.

### 3.6 Analysis of co-cited references

**Figure 8:**
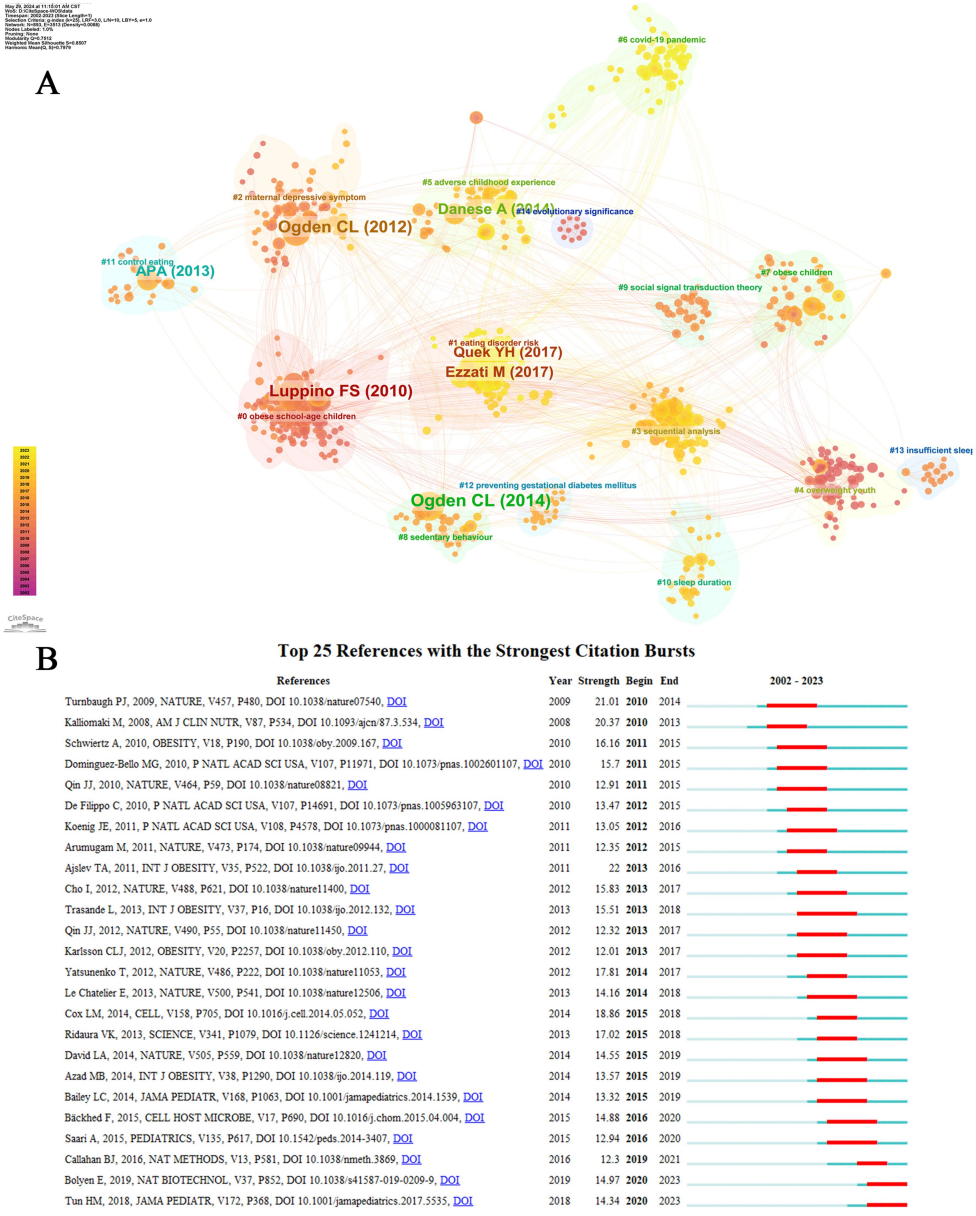
Reference analysis. **(A)** Cluster view of co-cited references in childhood obesity and gut microbiota. **(B)** Top 25 references with the strongest citation bursts.

Co-citations, or co-cited references, are citations that appear in multiple publications. These references form clusters, which may include foundational works and research groups, thereby indicating research trends and cutting-edge areas in a specific discipline (Lu et al., 2020). The retrieved literature was clustered and analyzed using CiteSpace into 15 clusters, as shown in Figure 8A. Notably, these clusters were well structured (Q = 0.7512) and highly credible (S = 0.7979). As can be observed from the figure, the publications in each cluster are closely related and harmonized with each other in a particular area. The cluster labeled as “obese school-age children” (Group 0) constitutes the largest proportion, followed by “eating disorder risk” (cluster 1), “maternal depressive symptoms” (cluster 2), the analytical methodology of “sequential analysis” (cluster 3), and the worrying issue of “overweight youth” (cluster 4). In the recent decades, there has been a tendency toward studying the correlation between childhood obesity and intestinal microbiota, which is predominantly skewed toward specific domains: Obese school-age children (group 1), Maternal depressive symptoms (group 3), Adverse childhood experiences (group 5), the global impact of the covid-19 pandemic (group 6), and the emergence of novel research on childhood obesity (group 7). Moreover, sleep duration (group 10) is also an aspect of immense interest. With regard to the duration of these studies, the primary focus was on “Obese school-age children” (group 0), “overweight youth” (group 4), and “obese children” (cluster 7). However, other pivotal clusters, such as “sedentary behavior” (cluster 8), the theoretical framework of “social single transaction theory” (cluster 9), practices aimed at “controlling eating” (cluster 11), measures for “preventing gestational diabetes mellitus” (cluster 12), “insufficient sleep” (cluster 13 and group 13), and the overarching issue of “the lack of a healthy diet” (cluster 14) continue to captivate researchers’ attention. The topic of “Evolutionary significance” (group 14) further broadens the scope of inquiry in this field.

In order to understand the most frequently cited literature in recent years, we conducted a comprehensive analysis of the top 25 references with the most substantial citation bursts (Figure 8B). The year of publication indicates the research’s vintage, while the intensity of the citation bursts reflects the level of attention garnered by researchers. The start and end points denote the duration during which the literature was frequently cited, corresponding to the conspicuous red section on the graph. With a cutoff date of 2023, the most recently cited works include Tun HM, 2018 (Intensity: 14.34, Time span: 2020–2023), Bolyen E, 2019 (Intensity: 14.97, Time span: 2020–2023), Callahan BJ, 2016 (Intensity: 12.3, Time span: 2019–2021), and numerous other notable contributions.

### 3.7 Analysis of keyword co-occurrence

**Figure 9:**
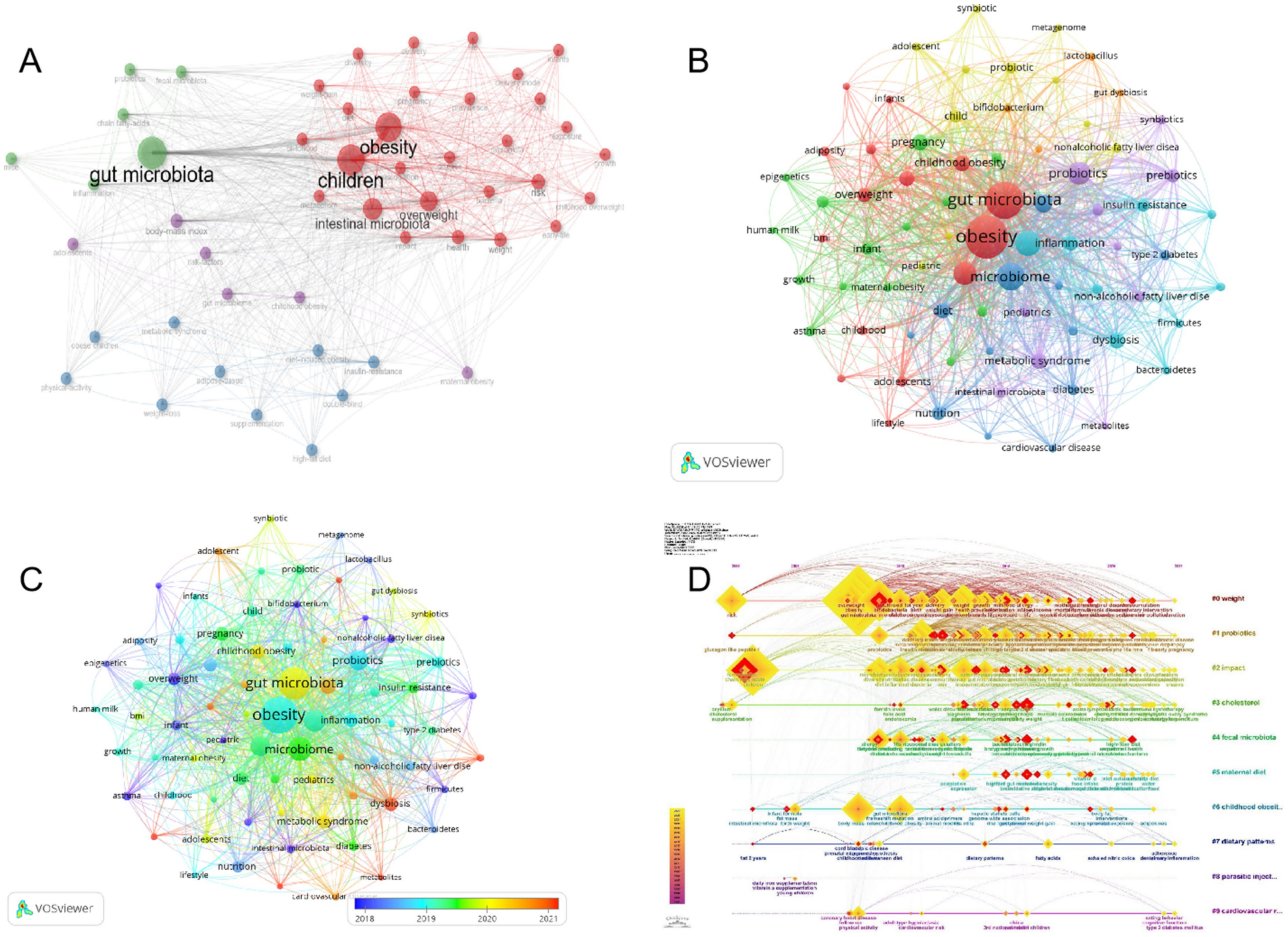
Author keywords analysis. **(A)** Keyword Co-Occurrence Network. **(B)** Timeline of keywords in the field of childhood obesity and gut microbiota. **(C)** Author Keywords Co-occurring Networks. **(D)** Temporal overlapping co-occurrence analysis network for author keywords, which enhances literature clustering through chronological expansion and identifies the initial appearance of each key term.

The Keyword Co-occurrence Network presents profound insights into the thematic tendencies within the realm of childhood obesity and gut microbiota. By scrutinizing the intricate associations between the keywords within the literature corpus and their respective weights, one is able to acquire a more profound comprehension of the intricate composition and intricate structure that characterize this scientific terrain. This approach offers a nuanced perspective into the intricate interplay of factors within this critical research area (Radhakrishnan et al., 2017). As depicted in Figure 9A, the terms “gut microbiota” and “children” are consistently highlighted by authors, suggesting their pivotal significance in the realm of research pertaining to childhood obesity and gut microbiota. Moreover, the inclusion of subject terms like obesity, intestinal microbiota, overweight, body-mass index (BMI), and health, further enables researchers to embark on a more extensive exploration of pertinent literature in this vital domain.

The evolution of keywords over time is clearly demonstrated in the overlay visual map shown in Figure 9B. Cluster 1 (red nodes, representing obesity and gut microbiota), cluster 2 (green nodes, representing pregnancy), cluster 3 (blue nodes, representing microbiome), cluster 4 (yellow nodes, representing child), cluster 5 (purple node, representing probiotics), cluster 6 (cyan blue, representing microbiota), and cluster 7 (orange node, representing *Bifidobacterium*). The temporal evolution of the keywords can be observed in the superimposed visual map shown in Figure 9C. The proximity of a node’s color to red suggests its recency, implying that these keywords may signify current and potential research hotspots. Notably, keyword nodes with hues near red, such as “dysbiosis,” “metabolites,” “high-fat diet,” “autism spectrum disorder,” and “cancer,” appear to delineate contemporary research frontiers. Furthermore, we employed the capabilities of CiteSpace for keyword analysis to generate an intriguing timeline chart that vividly illustrates the emergence and decline of research hotspots in childhood obesity and gut microbiota from 2002 to 2023 (Figure 9D). Between 2002 and 2010, high-impact keywords centered on gut microbiota, risk, fermentation, children, chain fatty acids, body mass, and gut microflora. The first cluster to emerge was cluster 1, with “risk” as the keyword, showcasing the historical discovery of obesity risk factors in children. Cluster 0 (weight), cluster1 (probiotics), cluster 2 (impact), cluster 3 (cholesterol), cluster 7 (dietary patterns), and cluster 9 (cardiovascular risk) are all integral components of our current study. A perusal of this chronological depiction reveals a distinct progression in research emphases, highlighted by the evolving keywords from 2002 to 2023. This temporal analysis sheds valuable insights into the emergence and waning of pivotal keywords, reflecting the dynamic shifts in scholarly pursuits within the discipline. The keywords within each cluster are systematically arranged along a horizontal timeline, from left to right, highlighting the chronological development and profound significance of each cluster’s contribution to the scholarly advancements in the realm of childhood obesity and gut microbiota.

### 3.8 Analysis of hotspots and trends in research

**Figure 10:**
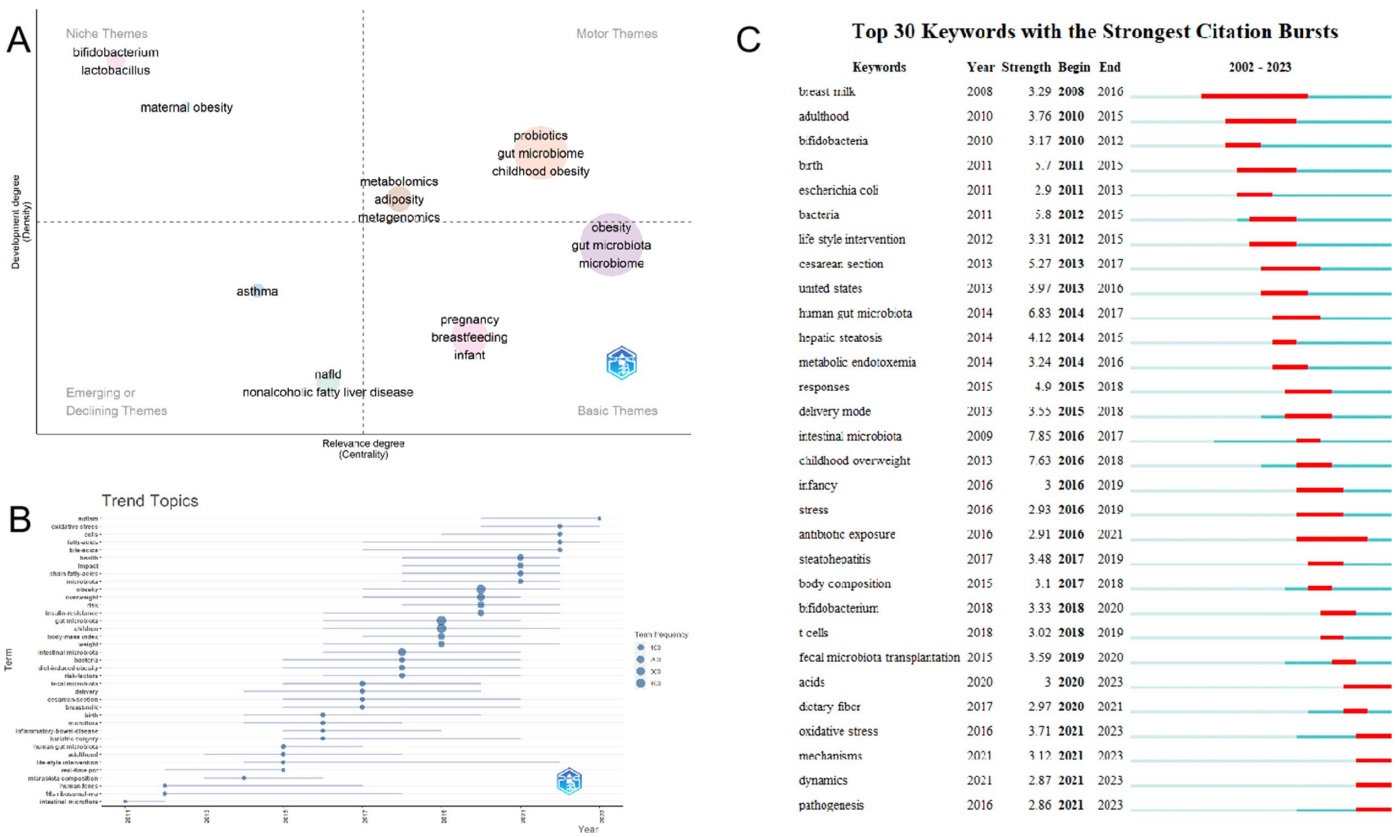
Hot Topics and Trends. **(A)** Thematic map. **(B)** Network map of keywords on burnout among nurses. **(C)** Trend of Topics over time.

After a thorough examination of the thematic map of the data, we crafted a detailed description of its underlying research trends and prominent hotspots. Centrality, in this context, serves as a paramount indicator of the significance of a theme within the research landscape, while density offers an insightful perspective on the evolution and progress of the theme itself. This comprehensive analysis enables us to gain a deeper understanding of the evolving dynamics in the research field (Hernández-González et al., 2023). Furthermore, the scale of each domain serves as a reflective prism, showcasing the immense volume of articles that have embraced the specific keyword as a fundamental pillar in their inquiries. The four quadrants illustrated in Figure 10A display differing levels of centrality and density. Within the domain under discussion, it has become apparent that there is profound exploration being undertaken regarding probiotics, the intricate gut microbiome, metabolomics, adiposity, and the intricate field of metagenomics. Central themes are indeed represented by these foundational elements. In this particular quadrant, a compelling need for further delving into the intricate connection between childhood obesity and the gut microbiome is evident. Moreover, pregnancy and breastfeeding are emerging as significant themes in this burgeoning research landscape.

In addition to this, CiteSpace has adeptly identified emerging keywords, as depicted in Figure 10B, highlighting the 30 most pertinent keywords exhibiting remarkable surges. Notably, topics such as “pathogenesis (2021–2023),” “dynamics (2021–2023),” “mechanisms (2021–2023),” “oxidative stress (2021–2023),” and “acids (2020–2023)” have consistently gained prominence in recent years, indicating a potential to remain at the forefront of research priorities in the foreseeable future. The keyword trend analysis presented in Figure 10C provides a captivating graphical illustration of evolving research priorities over the years. From 2011 to 2023, the focus of research has primarily revolved around “gut microbiota,” “children,” and “obesity,” with recurring keywords such as “gut microbiota,” “children,” and “obesity” standing out. Additionally, keywords like “overweight,” “intestinal microbiota,” and “fecal microbiota” have frequently appeared. Around 2018, the scope of research broadened to encompass aspects of gut microbiota associated with weight gain in children, with terms such as “gut microbiota,” “intestinal microbiota,” and “intestinal microbiota” taking center stage. Post-2021, “chain-fatty-acids,” “bile acids,” “fatty-acids,” “cells,” and “oxidative stress” emerged as frequently discussed and crucial terms. Notably, “autism” emerged as the most significant term in 2023, suggesting its potential to become a current research hotspot in the realm of studying childhood obesity and gut microbiota.

## 4 Discussion

Childhood obesity is a burgeoning health issue and poses a significant challenge to global public health (Fiore et al., 2023). Children’s obesity is associated with the gut microbiota, which is further supported by the Mendelian randomization study (Li Q. et al., 2023). In order to summarize global publications from 2002 to 2023 regarding childhood obesity and gut microbiota, we conducted a comprehensive analysis. The tools CiteSpace, VOSviewer, and R-bibliometrix were utilized to analyze a dataset of 1,384 articles related to the topic of childhood obesity and gut microbiota within the Web of Science. From this analysis, we evaluated spatial and temporal distributions, author contributions, core articles, as well as research hotspots and frontiers in this field. This provided a significant stimulus for enhancing our understanding of the relationship between childhood obesity and gut microbiota.

### 4.1 General information research development and contributions

In the past 3 years, global publications on childhood obesity and gut microbiota have shown a significant increase. The annual trends in publications have exhibited substantial fluctuations over time, including both upward and downward phases. These phases may be attributed to fragmentation, low-quality research, changes in research direction, and potentially unaddressed areas.

### 4.2 International collaboration among states, institutions, and authors

In this paper, we visualize the international collaboration in childhood obesity and gut microbiota research at three levels: national, institutional, and author. In terms of publications and citations, the United States dominates the research community. As for institutions, Canada is a major contributor to research on childhood obesity and gut microbiota, with four of the top 10 institutions in terms of number of publications coming from Canada. Seppo and Isolauri, Erika are the two authors with the highest number of publications and also the highest number of citations, demonstrating their profound impact on childhood obesity and gut microbiota research.

### 4.3 The most influential journal and co-cited reference

To reveal the importance of interdisciplinary research, we performed a visual analysis of academic journals in this field. According to an overlap analysis of cited journals, PLoS One and PNAS USA are the most cited journals in the field, emphasizing the interconnections between them. Literature co-citation cluster analysis deepened the knowledge structure of childhood obesity and intestinal microbiota research, focusing on intestinal microbiota. Furthermore, an analysis of the top 25 reference maps with the strongest citation bursts revealed that recent literature may indicate emerging trends in the relationship between childhood obesity and gut microbiota.

### 4.4 Hotspots and trends

The keyword co-occurrence analysis reveals the hotspots of research on childhood obesity and gut microbiota. The result reveals that “gut microbiota,” “obesity,” and “children” are the three predominant categories of research topics. A time line graph analysis of the 10 clusters was obtained using LSI, and these elements are all essential components of our ongoing research and serve as focal points. Notably, the following topics include “pathogenesis,” “dynamics,” “mechanisms,” “oxidative stress,” and “acids” have been consistently exploding in recent years, suggesting that in the future there is potential to remain at the forefront of research priorities, as well as providing a comprehensive understanding of hotspots and trends in the field of childhood obesity and gut microbiota.

Previous studies have primarily examined factors influencing the early development of the gut microbiota, such as host genetics, mode of delivery, diet, age, environment, and antibiotic use. These factors regulate the microbial community, potentially providing a foundation for novel therapeutic approaches to tackle childhood obesity (Pihl et al., 2016). Our study, which examines the emergence patterns of significant keywords, indicates that contemporary research primarily concentrates on several key areas.

Firstly, the gut microbiota plays a crucial role in the pathogenesis of childhood obesity, with recent studies highlighting its significant influence among the various contributing factors. A thorough 16S rDNA sequencing analysis disclosed that the gut microbiota composition in children experiencing varying degrees of obesity exhibited striking similarities (Wang et al., 2024b), its diversity notably varied from that observed in children with normal weight and those with a different composition (Li et al., 2024), for instance, the increased prevalence of *Actinobacteria* in the intestines of obese children (Wang et al., 2024b). PICRUSt analysis delved deeper into the metabolic discrepancies between obese and normal-weight children, revealing a diminished proportion of *Bacteroides* phylum and a corresponding augmentation in the *phyla Firmicutes*, resulting in a skewed ratio of *phyla Firmicutes* phylum to *Bacteroides* phylum. The composition of gut microbiota may indeed be a cornerstone in the development of obesity. With regard to the gut microbiota species that are currently under intensive scrutiny, researchers’ attention may be aptly directed toward them, potentially providing a robust scientific underpinning and theoretical backbone for the exploration of treatment modalities for childhood obesity.

The second aspect encompasses the period from the mother’s pregnancy to delivery, as well as the subsequent period of breastfeeding. Studies has demonstrated that the fetal gut microbiota initiates colonization before birth and undergoes continuous alterations during childbirth and breastfeeding. During pregnancy, the mother’s gut microbiota is transmitted primarily through her diet and the surrounding environment, which has an impact on the establishment of the fledgling fetal and neonatal microbiota (Faienza et al., 2024). Subsequent research has established a correlation between childhood obesity and certain maternal behaviors during pregnancy, including smoking (Kumar et al., 2014). Childhood obesity is associated with maternal smoking during pregnancy. Interestingly, the gut microbiota may partially mediate this association, as one study revealed that maternal smoking during pregnancy significantly escalated the abundance and diversity of the *Bacteroides* phylum, comprising thick-walled bacteria. The analogous behavior influences the formation of the gut microbiome in fetuses and neonates at early stages of development. This consequently results in epigenetic characteristics that predispose them to obesity as they grow and develop (Faienza et al., 2024). Therefore, smoking during pregnancy alters the microbiota and could be targeted for interventions to prevent childhood obesity. The World Health Organization (WHO) advocates for exclusive breastfeeding during the initial six months of an infant’s life (Olga et al., 2021). Breastfeeding significantly influences the health of the gut microbiota, as it is associated with elevated levels of beneficial bacteria such as *Bifidobacteria*, and decreased levels of potential pathogens (Maessen et al., 2020; Brockway, 2024). Breast milk can potentially prevent childhood obesity by supplying probiotics (Haddad et al., 2021). In breastfed children, *Lactobacillus plantarum* H-72 modulates the expression of genes related to energy metabolism, thereby regulating the structure of the intestinal microbiota and exhibiting potential anti-obesity effects. Furthermore, breast milk contains the complex sugars human milk oligosaccharides (HMOs), which help to develop the gut microbiome and immune system of the infant. A potential link between breastfeeding and a reduced risk of obesity has also been explored (Maessen et al., 2020). One study discovered that exclusive breastfeeding until the third month of age may lower the risk of childhood obesity later in life (Peng Y. et al., 2024). These findings suggest promising opportunities for future interventions aimed at preventing pediatric obesity in breastfed children (Liang et al., 2024).

Thirdly, various periods such as infancy, childhood, and adulthood are characterized by the colonization, maturation, and homeostasis of the gut microbiota. This process significantly impacts health during these stages by affecting immunity (Jielong et al., 2022). Early colonizers of infant guts have a lasting impact on the host’s health throughout life (Chawla et al., 2022). Dysbiosis of the microbiota during infancy has been linked to long-term health outcomes, including childhood obesity (Yelverton et al., 2023). The first 4 years of life are a critical period for microbiota maturation and a key window for establishing long-term developmental patterns of BMI (Reyna et al., 2022). Results from another two national birth cohort studies from France showed that the gut microbiota at 3.5 years of age was associated with BMI in late childhood, with a positive correlation between the *phyla Firmicutes* and Phylum *Bacteroidetes* (F/B) ratio at 3.5 years of age and a BMI z-score at 5 years of age (Toubon et al., 2024). This suggests that changes in the gut microbiota that may contribute to adult obesity begin in early childhood (Toubon et al., 2024). Previous investigations have indicated that imbalance in the gut microbiota during childhood can lead to an augmentation of bacterial taxa associated with obesity. Moreover, reestablishing equilibrium in the gut microbiota might mitigate obesity and its related comorbidities, such as hypertension, fatty liver, and type 2 diabetes mellitus (Akagbosu et al., 2022). A cohort study found that the gut microbiota is more influenced by obesity and related factors in childhood than in adulthood, and that its diversity is greater in childhood than in adulthood (Yu et al., 2023). The transition from childhood to adulthood necessitates increased attention to stabilizing the gut microbiota in childhood and early intervention to prevent adverse effects of alterations in the gut microbiota during childhood on adulthood. This is also crucial for timely intervention in early childhood obesity, improving child and adult health, and maintaining lifelong gut microbiota-associated health.

The fourth factor involves human gut microbiota, such as *Bifidobacterium* and *Escherichia coli* (*E. coli*), bacteria, and fecal microbiota transplantation (FMT). A macro-genomic study found that obese children had significantly higher levels of the bacterial pathogen *Campylobacter* compared to their non-obese counterparts (Li P. et al., 2023). Both *Bifidobacteria* and *E. coli* are significant in the human gut; the former is beneficial and has crucial physiological functions for health, while the latter helps maintain intestinal microecological balance. In a case–control study examining obesity in school-age children and its correlation with intestinal *E. coli* and *Bifidobacteria*, researchers discovered that obese children had lower levels of *Bifidobacteria* and higher levels of *E. coli* than normal children, suggesting an imbalance of intestinal microorganisms in obese children (Gao et al., 2015). Common gut microbes also include *phyla Firmicutes* and *Mycobacteria* phylum. A study comparing obese and non-obese Egyptian children and adults found that obesity was associated with changes in the composition of the fecal microbiota, particularly an increase in *phyla Firmicutes* and *Mycobacteria* phylum (Abdallah Ismail et al., 2011). Fecal microbiota transplantation (FMT) involves transferring the gut microbiota of a healthy individual into a patient to restore the patient’s gut microbiota balance and treat associated diseases (Oliva-Hemker et al., 2023). Some researchers observed in a randomized clinical trial involving obese children that, although FMT did not lead to weight loss, it reduced abdominal obesity and improved metabolic syndrome (Leong et al., 2020). These findings should be considered when developing strategies to control obesity and related comorbidities by modifying the gut microbiota.

Fifth, hepatic steatosis, steatohepatitis, and antibiotic exposure are factors to consider. Microbiome analysis has identified key bacteria in children with obesity and non-alcoholic fatty liver disease (NAFLD). Multi-omics and LC–MS/MS analyses, as well as microbial-derived metabolite results, have shown that *Enterococcus* spp. (species pluralis) are enriched in these children (Wei et al., 2024). The relationship between early antibiotic exposure and the development of the infant’s gut microbiome is complex, and these medications can significantly impact child health outcomes (Brockway, 2024). Moreover, antibiotic-altered gut microbiota may signal different immune effects that affect children’s health (Livanos et al., 2016). The widespread exposure of antibiotics in early childhood warrants further investigation.

Sixth, lifestyle interventions, dietary fiber, and stress also play a role. The environment can influence children’s physical condition; various lifestyles, dietary habits, sleep patterns, and physical activity levels can alter intestinal microbiota composition and impact physical health. Gut microbiota is crucial for the absorption, storage, and utilization of energy from food. Appetite regulation is complex, but stress significantly affects eating behavior. Acute stress typically decreases appetite, while chronic stress can lead to intense cravings for high-calorie, high-fat foods, increasing the risk of weight gain and obesity (Piątkowska-Chmiel et al., 2023). Diet is a key determinant of gut microbiota structure and function among multiple host-endogenous and host-exogenous factors (Zmora et al., 2019). This, in turn, regulates food intake as a key modulator of the gut microbiota-brain axis (Cryan et al., 2019). The gut-brain axis activates during food intake through gut hormone release, ultimately triggering the hypothalamus to release appetite suppressant-related hormones. This process potentially affects body weight by influencing appetite, storage, and energy expenditure (De Clercq et al., 2016). The intricate regulation of the gut-brain axis suggests a more profound complexity in the relationship between childhood obesity and gut microbiota, necessitating further study.

## 5 Limitation

This study has four limitations. First, it solely relied on the WoS database for article analysis, excluding relevant studies from other databases. Second, the literature review spans from 2002 to 2023, omitting a small number of studies from earlier years and 2024, as well as a limited amount of literature. Third, this study focused exclusively on reviews and articles, neglecting other relevant types of literature. Finally, VOSviewer and CiteSpace, which were used for the bibliometric analysis, have inherent limitations in that they are unable to analyze the full text of publications and are primarily applicable to English language literature, which introduces a selection bias leading to the potential oversight of certain information and the omission of more recently published articles.

## 6 Conclusion

This extensive study offers valuable insights into the global attention and evolving trends in childhood obesity and gut microbiota research spanning the period from 2002 to 2023. Firstly, a rigorous statistical analysis of the trends in these research domains reveals a substantial escalation in the number of publications over the years. Notably, the United States, China, and Canada have emerged as the leading nations in this field, with Canada boasting a significant academic presence, evidenced by the fact that a majority of the top 10 authors hail from this country. At the institutional level, the University of Copenhagen stands out as a leading institution.

Secondly, a comprehensive keyword co-occurrence and co-citation analysis of the childhood obesity and gut microbiota research provides a nuanced understanding of the research hotspots, emerging trends, and offers profound perspectives on the field’s development. This analysis serves as a window into the evolving research landscape.

Lastly, the utilization of VOSviewer visualization analysis has revealed the core journals and cross-collaborations within the domain of childhood obesity and intestinal microbiota research. This visualization technique provides researchers with a roadmap to comprehensively access the latest research findings and advancements in the field.

In summary, this paper presents a comprehensive overview of the current status and dynamic trends in childhood obesity and gut microbiota research, offering researchers profound insights into this vital research domain.

## Footnotes

^1^https://bibliometric.com/
